# Indolent enhancing spinal lesions mimicking spinal metastasis in pediatric patients with malignant primary brain tumors

**DOI:** 10.1038/s41598-022-05831-6

**Published:** 2022-02-02

**Authors:** Hsin-Wei Wu, Shih-Chieh Lin, Ching-Lan Wu, Kang-Lung Lee, Chia-Hung Wu, Shu-Ting Chen, Hsin-Hung Chen, Yi-Yen Lee, Yi-Wei Chen, Chih-Chun Wu, Ting-Rong Hsu, Feng-Chi Chang

**Affiliations:** 1 Department of Radiology, Taipei Veterans General Hospital, 201 Sec. 2, Shih-Pai Rd., Taipei, 11217 Taiwan; 2 School of Medicine, National Yang Ming Chiao Tung University, Taipei, Taiwan; 3 Department of Pathology and Laboratory Medicine, Taipei Veterans General Hospital, Taipei, Taiwan; 4 Institute of Clinical Medicine, National Yang Ming Chiao Tung University, Taipei, Taiwan; 5 Division of Pediatric Neurosurgery, Department of Neurosurgery, Neurological Institute, Taipei Veterans General Hospital, Taipei, Taiwan; 6 Department of Oncology, Taipei Veterans General Hospital, Taipei, Taiwan; 7 Department of Medical Imaging and Radiological Technology, Yuanpei University of Medical Technology, Hsinchu, Taiwan; 8 Department of Pediatrics, Taipei Veterans General Hospital, Taipei, Taiwan

**Keywords:** Cancer imaging, CNS cancer, Metastasis

## Abstract

Spinal metastasis from malignant primary brain tumors (MPBTs) in pediatric patients is rare and often appears as enhancing lesions on MRI. However, some indolent enhancing spinal lesions (IESLs) resulting from previous treatment mimic metastasis on MRI, leading to unnecessary investigation and treatment. In 2005–2020, we retrospectively enrolled 12 pediatric/young patients with clinical impression of spinal metastasis and pathological diagnosis of their spinal lesions. Three patients had MPBT with IESL, and 9 patients had malignant tumors with metastases. The histopathologic diagnosis of IESL was unremarkable marrow change. We evaluated their MRI, CT, and bone scan findings. The following imaging findings of IESL vs. spinal metastasis were noted: (1) IESLs appeared round/ovoid (3/3, 100%), whereas spinal metastasis appeared irregular (9/9, 100%) (P = 0.005); (2) target-shaped enhancement was noted in (3/3, 100%) vs. (0/9, 0%) of cases, respectively (P = 0.005); (3) pathologic fracture of the vertebral body was noted in (1/3, 33.3%) vs. (9/9, 100%) of cases, respectively (P = 0.045); (4) expansile vertebral shape was noted in (0/3, 0%) vs. (9/9, 100%) of cases, respectively (P = 0.005); (5) obliteration of the basivertebral vein was noted in (0/3, 0%) vs. (9/9, 100%) of cases, respectively (P = 0.005); and (6) osteoblastic change on CT was noted in (3/3, 100%) vs. (2/9, 22.2%) of cases, respectively (P = 0.034). IESL in pediatric patients with MPBT can be differentiated from metastasis based on their imaging characteristics. We suggest close follow-up rather than aggressive investigation and treatment for IESL.

## Introduction

Malignant primary brain tumors (MPBTs) may metastasize through several pathways: local invasion, cerebrospinal fluid (CSF), and hematogenous or lymphangitic routes. Early neuroaxial metastasis through CSF is common, occurring in up to 53.5% glioblastoma patients and in approximately 33% at the initial diagnosis of medulloblastomas^1,2^. To achieve complete tumor control, craniospinal radiotherapy of the whole spinal region plus chemotherapy is the standard treatment for pediatric MPBT with leptomeningeal seeding^3^. In contrast with neuroaxial metastasis, distant extraneural metastases are rare, ranging from 0 to 7% depending on the brain tumor type^4–7^. Most extraneural metastases occur after craniotomy or diversionary CSF shunting due to damage to the blood–brain barrier^8^. Common distant metastatic sites of pediatric brain tumors include bone (56.3%, especially at pelvis, femur and vertebrae), visceral organs (55.5%, mostly liver and lung), and lymph nodes (25.3%)^4^. Adjuvant treatment, such as chemoradiotherapy or radiotherapy, is favored for distant extracranial metastases.

MRI is the best imaging technique for diagnosing MPBT-associated spinal metastasis (SM) and leptomeningeal seeding^9^. An enhancing lesion of the spine on contrast-enhanced T1WI with/without adjacent soft tissue lesions is usually the crucial finding of SM. However, some indolent enhancing spinal lesions (IESLs) on MRI may mimic SM^10,11^. Histological expression of the IESL is usually unremarkable with no malignant cells. The IESL may be the sequelae of previous radiotherapy, chemotherapy, stem cell transplantation, and medications, such as corticosteroids, aromatase inhibitors, and bisphosphonates^10,11^. Without an accurate diagnosis, pediatric patients with IESL may receive unnecessary invasive investigation and adjuvant treatment.

This retrospective study assessed the imaging findings of pathologically proven IESL of MPBT and SM of malignant tumors in pediatric and young patients, aiming to identify the differentiating features of IESL from SM and thus to improve therapeutic outcomes.

## Methods

### Patients

From 2005 to 2020, 12 pediatric or young patients with enhancing spinal lesions on MRI were enrolled (Table 1). All patients had a presumptive diagnosis of SM based on enhancing spinal lesions on MRI. The final histopathological diagnoses were 3 pediatric patients with MPBT with IESL and 9 pediatric/young patients with malignant tumors with SM. We recruited 9 young patients with SM under 30 years of age because there was no pediatric patient with brain tumor and SM identified on spinal MRI in our institute from 2005 to 2020. We thoroughly reviewed the patients’ clinical data, cancer treatment (radiotherapy, chemotherapy, and peripheral blood stem cell transplantation), surgical records, biopsy methods, and pathology results.

**Table 1:** Demographic characteristics of the 12 patients with enhancing spinal lesions on MRI.

Characteristic	IESL (N = 3)	SM (N = 9)	P value
Female sex—no. (%)	1 (33.3)	7 (77.8)	0.24
Age at the initial diagnosis of primary tumor—year^†^	11.0 ± 0.0 (11–11)	18.0 ± 9.8 (2–29)	0.28
Age at the presence of spinal lesions—year^†^	17.0 ± 9.5 (11–28)	19.0 ± 9.6 (3–29)	0.60
Interval between the initial diagnosis of primary tumor and presence of spinal lesions—month^†^	75.3 ± 113.3 (5–206)	8.1 ± 11.8 (2–31)	0.19
**Primary tumor type**			**0.009**
Brain tumor—no. (%)	3 (100)	0 (0)	
Musculoskeletal malignancy—no. (%)	0 (0)	5 (55.6)	
Lung cancer—no. (%)	0 (0)	1 (11.1)	
Breast cancer—no. (%)	0 (0)	2 (22.2)	
Neuroendocrine tumors of unknown origin—no. (%)	0 (0)	1 (11.1)	
**Systemic treatment of primary malignant tumors**			
Chemotherapy—no. (%)	3 (100)	8 (88.9)	1.00
Peripheral blood stem cell transplantation (PBSCT)—no. (%)	1 (33.2)	2 (22.2)	1.00
Whole spine irradiation—no. (%)	3 (100)	0 (0)	**0.005**
**Pathology examination method**			0.18
Computed tomography (CT)-guided biopsy—no (%)	3 (100)^‡^	3 (33.3)	
Surgery—no (%)	0 (0)	6 (66.7)	
Follow-up period after the initial diagnosis of primary tumor—month^†^	86.7 ± 116.4 (5–220)	32.8 ± 26.0 (2–83)	0.86
Progression or recurrence of the primary tumor—no (%)	3 (100)	5 (55.6)	0.49
Progression of the spinal lesions on magnetic resonance image (MRI)—no (%)	3 (100)	6 (75)^§^	1.00
Survival—no (%)	1 (33.3)	0 (0)^¶^	0.27

*IESL indolent enhancing spinal lesions, *SM* spinal metastasis.

^†^Data are expressed as the mean ± standard deviation (range).

^‡^One patient had undergone CT-guided biopsy twice, both of which identically disclosed IESLs.

^§^One patient in the SM group had no follow-up image for the bone lesions and was thus excluded from the calculation.

^¶^One patient in the SM group was lost to follow-up after 50 months of treatment in our hospital; therefore, she was excluded from the survival rate calculation.

### Imaging studies

Several imaging studies were performed in all patients given the clinical impression of SM on MRI and CT (Table 2). A Tc99m bone scan was also performed in 11 of the 12 patients. All spinal lesions were not seen initially but identified in the sequential follow-up images. The spinal MRI sequences included axial and sagittal T1/T2-weighted imaging (T1WI/T2WI) and contrast-enhanced fat-suppressed T1WI. In contrast-enhanced T1WI, we analyzed the enhancing spinal lesions by their location (cervical, thoracic, lumbar, sacral, or whole spine), number, shape (round, ovoid, or irregular), and margin (well-defined or ill-defined). Some typical findings of SM were also evaluated, including expansile change with convex border, pathological fracture of the involved vertebral body, posterior element involvement, paraspinal soft tissue, epidural soft tissue lesion with a “draped curtain sign”, obliteration of the basivertebral vein, and presence of leptomeningeal seeding^9,12^. We also recorded the enhancing spinal lesions that abutted a vertebral endplate or involved the corner of a vertebral body. We further analyzed the signal intensity of the lesions on T1WI, T2WI and contrast-enhanced T1WI compared with paraspinal muscle on MRI. A special “target enhancement” pattern of the lesions on contrast-enhanced T1WI was evaluated.

**Table 2:** Image features of the 12 patients with enhancing spinal lesions on MRI.

Image features	IESL (N = 3)	SM (N = 9)	P value	Odds ratio
**Magnetic resonance image (MRI)**				
Lesion location			0.51	
Confined to T/L-spine—no. (%)	0 (0)	3 (33.3)		
Whole spine—no. (%)	3 (100)	6 (66.7)		
Lesion number			0.12	
1–10—no. (%)	0 (0)	5 (55.6)		
11–20—no. (%)	3 (100)	4 (44.4)		
21–30—no. (%)	2 (66.7)	2 (22.2)		
$\ge$ 31—no. (%)	0 (0)	1 (11.1)		
Irregular lesion shape	0 (0)	0 (100)	**0.005**	
Ill-defined lesion margin	1 (33.3)	9 (100)	**0.045**	
Abutting endplate—no. (%)	2 (66.7)	9 (100)	0.25	
Corner involvement—no. (%)	3 (100)	7 (77.8)	1.00	
Posterior element involvement—no. (%)	2 (66.7)	8 (88.9)	0.46	0.25
Target-shaped enhancement—no. (%)	3 (100)	0 (0)	**0.005**	
Pathologic fracture—no. (%)	1 (33.3)	9 (100)	**0.045**	
Epidural soft tissue—no. (%)	0 (0)	8 (88.9)	**0.018**	
Paraspinal soft tissue—no. (%)	0 (0)	7 (77.8)	**0.045**	
Expansile vertebral shape– no. (%)	0 (0)	9 (100)	**0.005**	
Obliteration of basivertebral vein—no. (%)	0 (0)	9 (100)	**0.005**	
T1WI^‡^			–	
Hyperintensity or isointensity—no. (%)	0 (0)	0 (0)		
Hypointensity—no. (%)	3 (100)	9 (100)		
T2WI			**0.021**	
Hyperintensity—no. (%)	1 (33.3)^†^	6 (66.7)		
Isointensity—no. (%)	0 (0)	3 (33.3)		
Hypointensity—no. (%)	2 (66.7)	0 (0)		
Contrast-enhanced T1WI			–	
Enhancement—no. (%)	3 (100)	9 (100)		
No enhancement—no. (%)	0 (0)	0 (0)		
Leptomeningeal seeding—no. (%)	3 (100)	2 (22.2)	**0.045**	
**Computed tomography (CT)**			**0.034**	
Osteoblastic—no. (%)	3 (100)	2 (22.2)		
Osteolytic—no. (%)	0 (0)	6 (66.7)		
Mixed—no. (%)	0 (0)	1 (11.1)		
**Tc99m bone scan**			0.055	
Abnormal uptake—no. (%)	1(33.3)	8 (100)^‡^		
No abnormal uptake—no. (%)	2 (66.7)	0 (0)		

*IESL indolent enhancing spinal lesions, *SM* spinal metastasis, *MRI* magnetic resonance image, *T1WI* T1-weighted image, *T2WI* T2-weighted image.

^†^One IESL patient had lesions with both high and low signal intensity on T2WI. Given that most lesions exhibited T2 hyperintensity, this patient was assigned to this group.

^‡^Tc99m bone scan was not performed in one SM patient.

In the CT scan, we analyzed the lesions with either osteoblastic or osteolytic changes and the presence of pathologic fractures. Bone lesions with abnormal uptake on Technetium-99 m methylene diphosphonate (Tc99m-MDP) bone scans were also documented.

### Pathologic diagnoses

**Figure 1:**
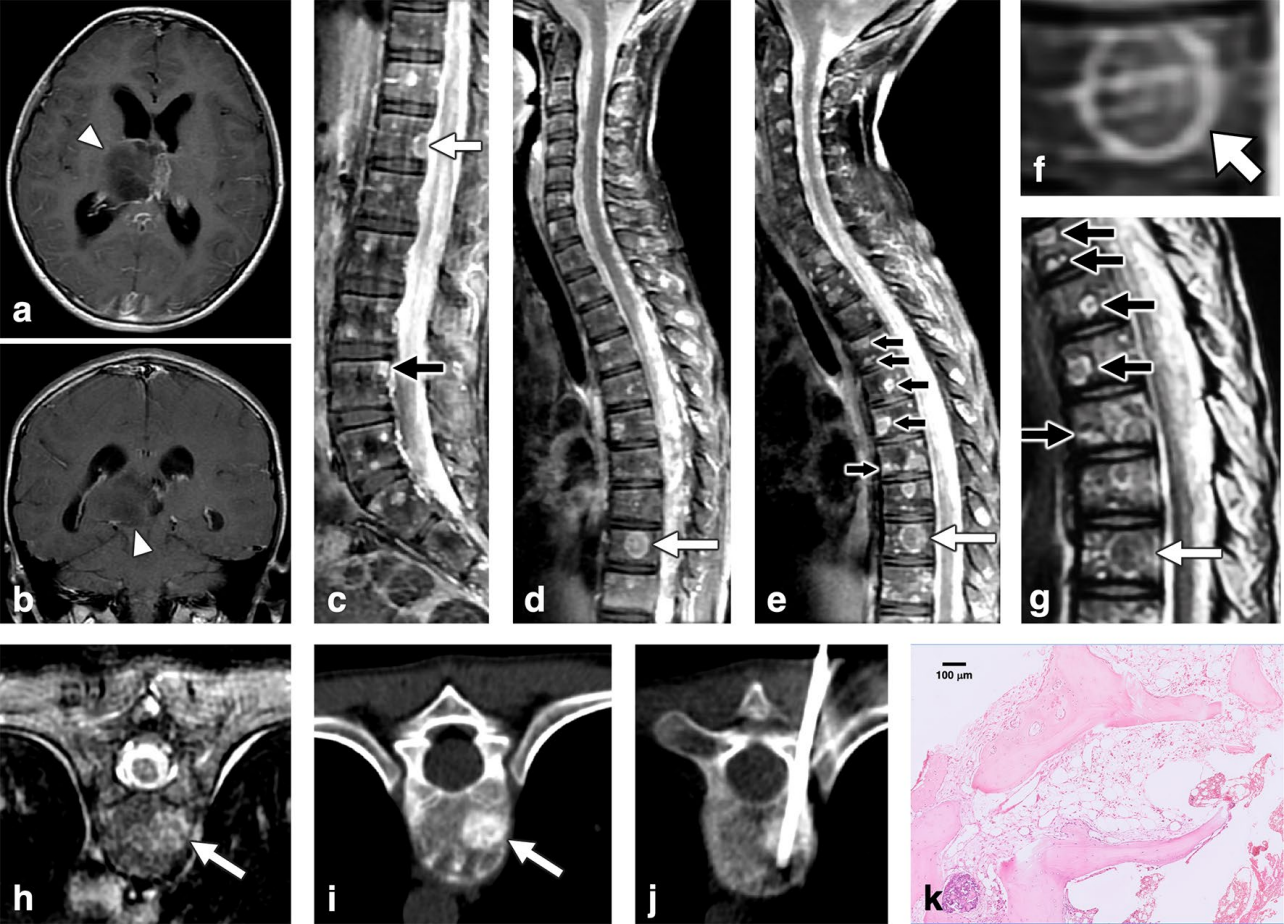
Indolent enhancing spinal lesion (IESL)—Case 1. An 11-year-old girl with anaplastic astrocytoma (2016 WHO grade III) over the right thalamus (**a**,**b**) was treated with partial tumor removal, radiotherapy, and chemotherapy. Four months after the surgery, sagittal contrast-enhanced T1WI revealed diffuse leptomeningeal seeding and multiple enhancing lesions over the whole spine (**c**,**d**). Target enhancement (**d**, arrow), ring enhancement (**c**, white arrow) and corner involvement (**c**, black arrow) were present. In the follow-up MRI 6 months after brain tumor surgery, sagittal contrast-enhanced T1WI of the cervical to middle thoracic spine revealed progression of the lesions (**e**). A target-enhancing pattern was specified at the T10 level (**d**,**e**, white arrow; magnified in **f**), which appeared hypointense on T2WI (**g**, white arrow). Coexisting lesions with ring enhancement on T1WI (**e**, black arrow) and hyperintensity with a “double line sign” on T2WI were also noted (**g**, black arrow). The T10 target-enhancing lesion on axial contrast-enhanced T1WI (**h**) revealed osteoblastic changes on CT scan (**i**). The first CT-guided biopsy disclosed no histologic evidence of malignancy (**j**). One week later, the second CT-guided biopsy revealed hypocellular marrow tissue and bone dust (H&E staining) (**k**).

**Figure 2:**
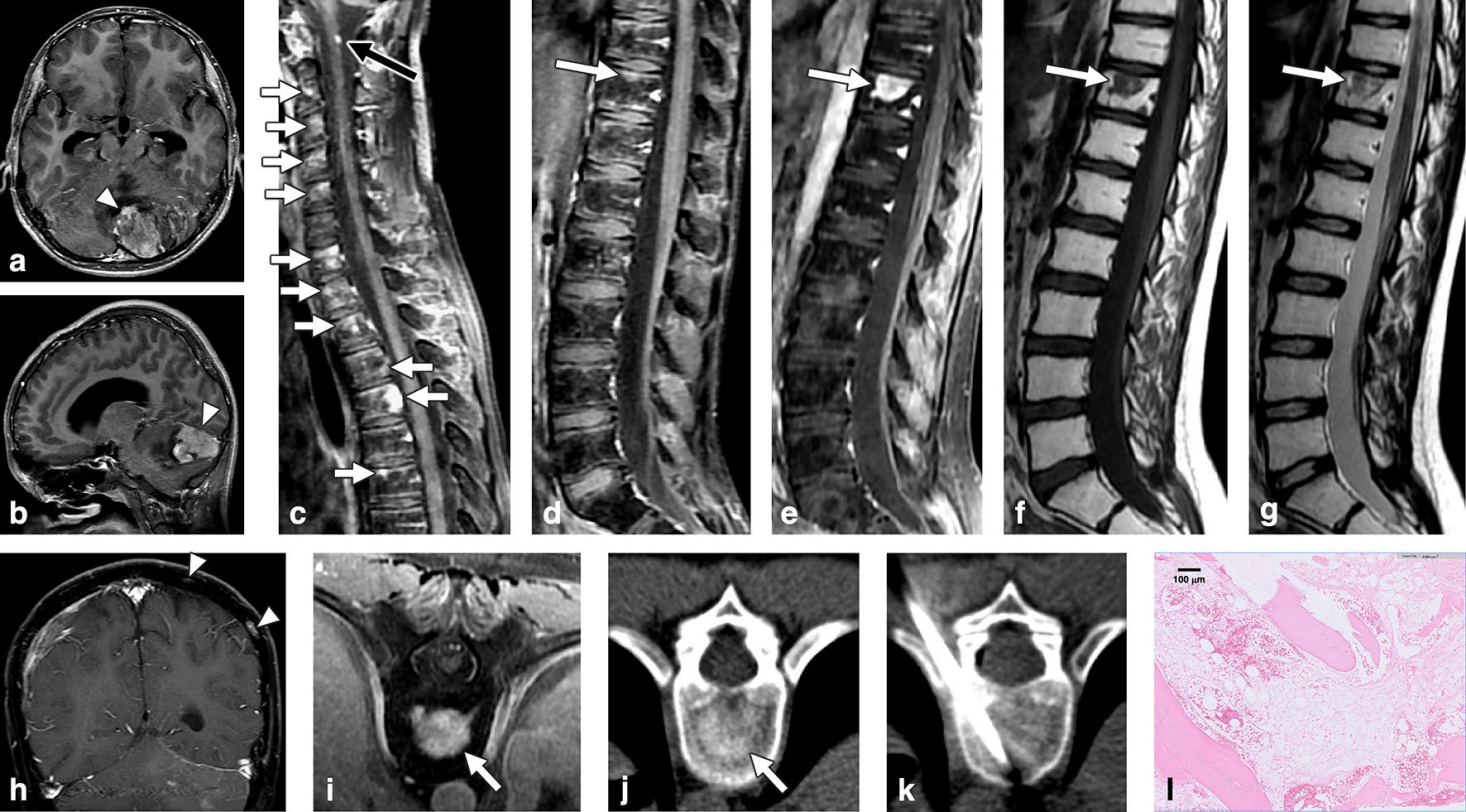
Indolent enhancing spinal lesion (IESL)—Case 2. An 11-year-old boy with medulloblastoma of the cerebellum (**a**,**b**) had undergone surgical resection and chemoradiotherapy. Sagittal contrast-enhanced T1WI revealed multiple enhancing foci in the cervical to thoracic spine 15 months after the diagnosis (**c**,**d**). Focal leptomeningeal seeding was noted at the C1 level (**c**, black arrow). In the follow-up MRI 33 months after brain tumor surgery, sagittal contrast-enhanced T1WI revealed significantly progressive changes in the lesion at vertebrae T11 with a target enhancement pattern (**e**, arrow). The lesion exhibited hypointensity on T1WI (**f**) and hypointensity on T2WI (**g**). Several enhancing lesions were also present at the temperoparietal skull bone of the brain MRI (**h**). On axial images, the T11 lesion had strong enhancement on contrast-enhanced MRI T1WI (**i**) and osteoblastic changes on the CT scan (**j**). CT-guided biopsy of the T11 lesion showed mild marrow fibrosis, adipose tissue filled in the marrow spaces, and scattered hematopoietic cells (H&E staining) (**k**,**l**).

**Figure 3:**
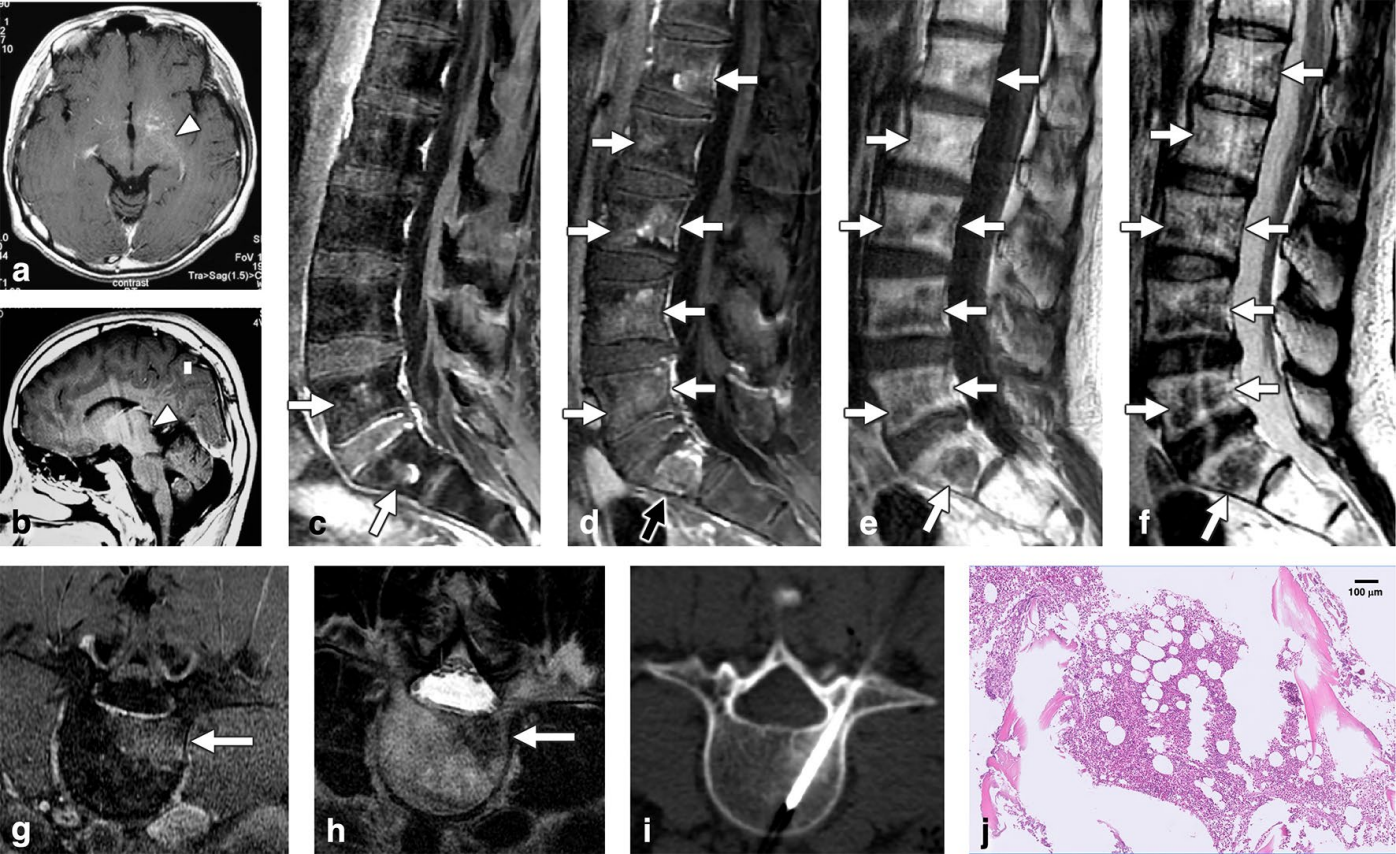
Indolent enhancing spinal lesion (IESL)—Case 3. An 11-year-old boy had a germ cell tumor over the thalamus to the hypothalamus (**a**,**b**). He had completed radiotherapy and chemotherapy. Seventeen years after the initial diagnosis, follow-up spinal MRI revealed enhancing lesions in the lumbosacral region on sagittal contrast-enhanced T1WI (**c**, arrow). Progressive changes in the lesions over the whole spine were observed one year later (**d**–**f**). These lesions appeared well enhanced on contrast-enhanced T1WI (**d**,**g**) with hypointensity on T1WI (**e**) and T2WI (**f**,**h**). A target enhancement pattern was also identified (**d**, black arrow). CT-guided biopsy of the L4 lesion revealed nearly normal hematopoiesis (H&E staining) (**i**,**j**).

Under the clinical impression of SM, pathological assessment of the suspected metastatic bone lesions was performed to confirm the diagnosis. Tissue was obtained either by CT-guided biopsy or surgical biopsy. For CT-guided biopsy, a single long-core bony specimen was obtained with an 11-gauge Jamshidi™ bone marrow biopsy needle after localization (Figs. 1, 2, 3). Surgical biopsy was performed by wide resection of the lesion via an open technique for decompressing the extradural compression. Immunohistochemical staining was used in histological studies to detect metastasis.

### Statistical assessment

All statistical analyses were performed with IBM^®^ SPSS^®^ statistics subscription. Continuous variables were summarized as the mean values with standard deviations; P values were calculated with the Mann–Whitney U test. Categorical variables were summarized as counts and percentages. P values were calculated with Fisher’s exact test in two categorical variables and via likelihood ratio for the variables with more than 3 categories. Patients with missing data for a variable were excluded from the analysis of the specified variable. All reported P values are two-sided. P values less than 0.05 were considered statistically significant.

### Approval for human experiments

This retrospective study was approved by the institutional review board of Taipei Veterans General Hospital (IRB-TPEVGH No.: 2021–07-005BC). All methods were performed in accordance with relevant guidelines and regulations. Written informed consent was obtained from each patient or their families to perform the MR examinations and the interventional procedures.

## Results

### Demographic features

The characteristics of the 3 IESL patients and the 9 SM patients are provided in Table 1. Tissue examination of 2 of the IESL cases revealed hypocellular marrow, fibrosis, and adipose tissue replacement; the other IESL case had normal marrow with nearly normal hematopoiesis. One patient with IESL had undergone repeated CT-guided biopsy in different bone lesions, disclosing similar pathology with no malignant cells. The two groups did not differ in age, sex, primary tumor origin, systemic treatment (chemotherapy, radiotherapy, peripheral blood stem cell transplantation), or biopsy methods used. The time interval from the diagnosis of primary malignant tumors to the presence of spinal lesions was 75.3 ± 113.3 (5–206) months in the IESL group and 8.1 ± 11.8 (2–31) months in the SM group (P = 0.19). No fluctuation was observed in the complete blood count of all 12 patients at the time the spinal lesions were discovered. All patients in both groups had received systemic chemotherapy for the primary malignant tumor. The 3 pediatric IESL patients underwent craniospinal radiotherapy as the management of MPBT with leptomeningeal seeding. During the follow-up period, all 3 IESL patients and 6 of the 8 SM patients (75%) exhibited progressive changes in the spinal lesions on MRI (P = 1.0) (Figs. 1, 2, 3). In addition to IESL, new scattered enhancing lesions were present over the skull of all 3 patients with MPBT in their follow-up brain MRI (Fig. 2H).

### Imaging findings

**Figure 4:**
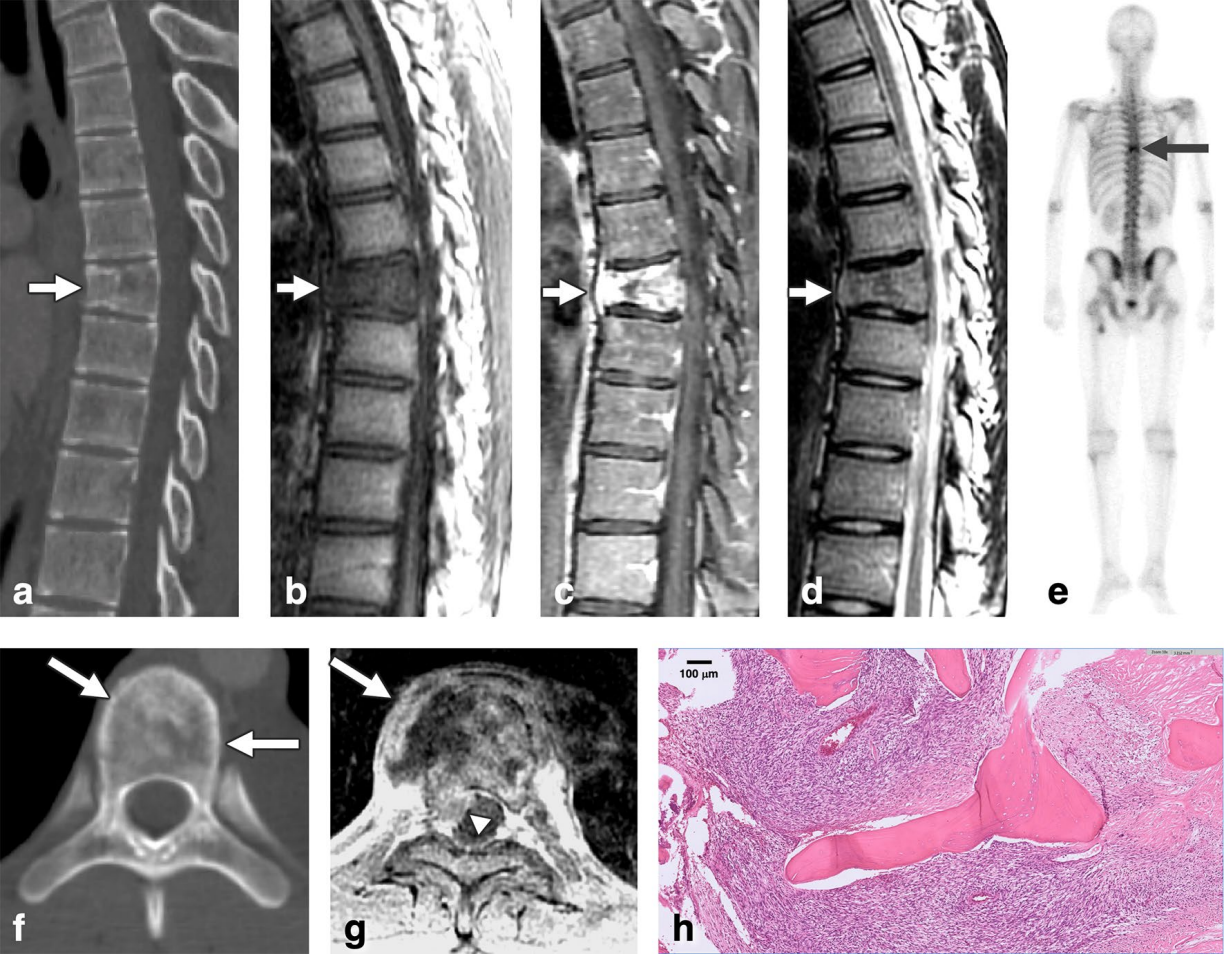
A case of spinal metastasis (SM). A 15-year-old male with synovial sarcoma over the left knee and pulmonary metastasis had undergone tumor resection and radiotherapy. Twenty-two months after the initial diagnosis, follow-up chest CT revealed an osteolytic lesion at the T7 spine with partial collapse of the vertebral body (**a**). MRI revealed hypointensity on T1WI (**b**), strong enhancement on contrast-enhanced T1WI (**c**), and isointensity on T2WI (**d**). Tc99m bone scan revealed avid uptake over T7 (**e**, arrow). The T7 lesion appeared osteolytic on axial CT image (**f**). Axial contrast-enhanced MRI T1WI at the T7 level revealed enhancing lesions with expansile changes, paraspinal soft tissue (**g**, arrow), epidural soft tissue (**g**, arrowhead), and obliteration of the basivertebral vein. Bone metastasis was histologically proven by T7 corpectomy (H&E staining) (**h**).

Image features of the bone lesions for the IESL and SM are listed in Table 2. The IESLs were round or ovoid (3/3, 100%), whereas all SM lesions appeared irregular (9/9, 100%) (P = 0.005). Ill-defined lesion margins were noted in 1 of the 3 IESLs (33.3%), whereas all true metastases had ill-defined margins (100%) (P = 0.045) (Figs. 3, 4).

For the associated findings of the vertebral lesions, SM had a significantly higher incidence of pathological fracture than IESL (9/9 [100%] vs. 1/3 [33.3%], P = 0.045), paraspinal soft tissue lesions (7/9 [77.8%] vs. 0/3 [0%], P = 0.045), epidural soft tissue (8/9 [88.9%] vs. 0/3 [0%], P = 0.018), expansile vertebral shape (9/9 [100%] vs. 0/3 [0%], P = 0.005), and obliteration of the basivertebral vein (9/9 [100%] vs. 0/3 [0%], P = 0.005) (Fig. 4). The MPBT patients with IESL had more leptomeningeal seeding compared with patients with SM during their disease courses (3/3 [100%] vs. 2/9 [22.2%], P = 0.045) (Figs. 1, 2).

For MR signals, IESL had significantly more hypointense signals on T2WI compared with SM (2/3, [66.7%] vs. 0/0, [0%], P = 0.021). No difference in signal intensity was observed on T1WI and contrast-enhanced T1WI between the two groups. A target enhancement pattern of the large lesions on contrast-enhanced T1WI was present in all 3 IESL patients (3/3, 100%) (Figs. 1, 2, 3)**,** but this pattern was not observed in the SM group (0/9, 0%) (P = 0.005).

In the CT scans, all IESLs had osteoblastic changes (3/3, 100%) (Figs. 1, 2, 3), whereas only 2 SMs had osteoblastic changes (2/9, 22.2%) (P = 0.034). For the 11 of our 12 patients who underwent bone scans, a trend of a higher incidence of uptake was noted in SM patients (8/8, 100%) (Fig. 4) compared with IESL patients (1/3, 33.3%) (P = 0.055).

## Discussion

**Figure 5:**
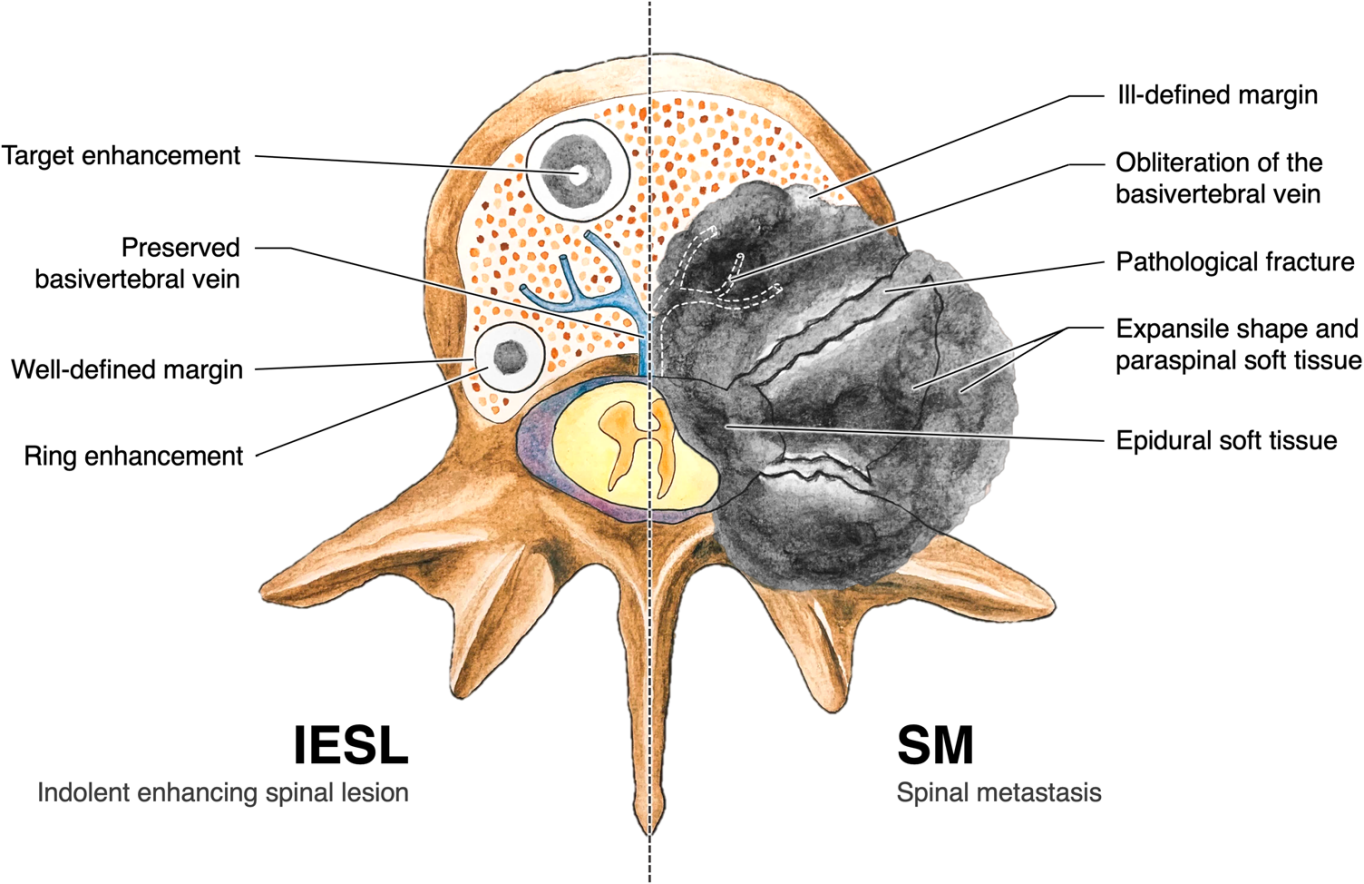
Indolent enhancing spinal lesion versus spinal metastasis. Comparison of the image characteristics of IESL and SM. IESLs tend to (1) be round/ovoid and well-defined; (2) exhibit an osteoblastic appearance on CT; (3) display target-shaped enhancement on contrast-enhanced MRI; (4) have preserved basivertebral veins; and (5) lack vertebral pathological fractures, paraspinal soft tissue, and expansile vertebral shapes.

Enhancing lesions on spinal MRI is a common finding in patients with malignant tumors and spinal metastasis. Treatment-related vertebral enhancing lesions have seldom been reported, especially in pediatric patients^10,11,13^. Our study compared the imaging findings between treatment-induced IESL and true metastasis to the spine (Fig. 5). The main imaging characteristics of IESL were (1) round/ovoid and well-defined shape; (2) osteoblastic appearance on CT; (3) target-shaped enhancement on contrast-enhanced MRI T1WI; (4) hypointensity on MRI T2WI; (5) preserved basivertebral vein; and (6) lack of vertebral pathological fracture, paraspinal soft tissue, and expansile vertebral change. An accurate diagnosis of these enhancing lesions on MRI helps to prevent unnecessary invasive investigation and to promote appropriate management.

SM from extracranial malignant tumors in pediatric patients usually presents as inhomogeneous bone lesions in the CT scan, which can be either lytic, sclerotic, or mixed^14,15^. Typical MRI characteristics of SM include expansile changes of the involved vertebrae, pathological fractures, pedicle or posterior element involvement, and paraspinal/epidural masses^9,14,16,17^. These lesions are often hypointense on TIWI, hyperintense on T2WI, and exhibit partial or marked postcontrast enhancement^14,15,18^. A hypointense lesion with a hyperintense rim on T2WI, i.e., “the halo sign”, is also an indicator of metastasis^9^. Metastatic lesions generally have avid uptake on bone scans^15^. In contrast, pediatric MPBT with distant extraneural SM is rare. MPBT with SM has imaging features similar to those of SM from extracranial malignant tumors^15,16,19–23^.

In our 3 IESL cases, neither malignant cells nor active inflammatory processes were found on pathology examination. In addition, these lesions were noted in the serial follow-up images but were not observed in the initial diagnosis of MPBT. These results suggest that IESL is likely a delayed response to the clinical treatment of MPBT with leptomeningeal seeding^10,11,13^. In the acute phase after radiotherapy and chemotherapy (within 1–2 weeks), cellular depletion and marrow edema occur with increased signal intensity on T2WI. Subsequently, fatty replacement and fibrosis occurs with disappearance of the red marrow. MRI then shows heterogeneous hyperintensity on T1WI, which is consistent with the presence of predominantly fat marrow. After 3–6 weeks of treatment, red marrow occasionally regenerates. The red marrow foci are hypointense on T1WI and T2WI with variable postcontrast enhancement^11,13,24,25^. Radiation-induced hematopoiesis is often patchy or band-like within the radiation portal; in contrast, hematopoiesis induced by chemotherapy, granulocyte-colony stimulating factor treatment, or hematopoietic stem cell transplantation is often diffuse, presenting as multifocal regenerating foci^11,13,24,26^. In the Tc99m bone scan, hematopoietic marrow usually has no abnormal uptake, but increased uptake has been reported due to high osteoblastic activity via hematopoietic cell proliferation^25,27^. Our IESL patients had predominantly hypointense signals on T1WI and T2WI with postcontrast enhancement on MRI, and 2 of 3 patients had no uptake in the bone scan. These findings are similar to those of red marrow regeneration. However, active hyperplastic hematopoiesis was not evident in any of the pathology specimens. Therefore, we suggest that hematopoiesis is a contributing factor but cannot completely explain the mechanism of IESL.

The other possible explanation for IESL of pediatric MPBT is bone marrow ischemia/necrosis. Marrow fibrosis is induced by radiotherapy and chemotherapy, which may lead to marrow ischemia and necrosis^28–30^. Radiation-induced marrow necrosis is generally localized within the radiation portal, i.e., osteoradionecrosis, which could have occurred in our patients because they were treated with whole spine irradiation for leptomeningeal seeding^11^. Typical imaging characteristics of spinal necrosis are discrete, well-defined, nonexpansile lesions, which lack soft tissue masses^31^. Osteonecrosis/infarction typically has a sclerotic appearance on CT scan, indicating calcification of the necrotic tissue or osteoblastic repair of the focal ischemic insult^18,30^. On MRI, these lesions often appear hypointense on T1WI and hyperintense on T2WI, which may become hypointense in late disease stages^18,31,32^. The “double line sign” is an image feature of early avascular necrosis and consists of a high-signal inner line representing hyperemic granulation and a low-signal outer parallel rim representing sclerotic bone on T2WI^13,32^. The layer of granulation tissue between necrotic and viable bone appears as rim enhancement in contrast-enhanced images^32^. One of our IESL patients had hyperintense spinal lesions with a “double line sign” on T2WI and ring enhancement on postcontrast T1WI (Fig. 1E,G), which is compatible with the common finding of bone infarction/necrosis^13,32^. The Tc99m bone scan demonstrates variable uptake in bone infarcts and no uptake in the necrotic regions^18,33^. In 2 of our 3 IESL patients, no uptake was evident on the bone scan, which is compatible with ischemia/necrosis or hematopoiesis^18,25,33^. However, some hot spots were observed in 1 IESL patient’s scan; this finding may be related to the uncommon presentation of bone infarct or focal hematopoiesis^27,33^. In contrast, all SM patients had significantly increased uptake in the bone scan.

In our study, the imaging presentation of all IESLs was mostly compatible with bone infarction/necrosis. Irradiation-induced cellular depletion and marrow fibrosis have been reported to progressively worsen over time, especially after 6 months of radiotherapy, which might explain the progressive change in our IESL^28^. Therefore, we favor a dynamic marrow response with fibrosis and delayed ischemic/necrotic bone insult as the major mechanism of IESL in pediatric MPBT patients who were treated with craniospinal irradiation and chemotherapy. This view is compatible with the histologic findings of hypocellular marrow with fat replacement and fibrosis in 2 of our 3 IESL patients. The pathological finding in the other IESL patient was nearly normal hematopoiesis, which can be explained by the combination of early infarction followed by late marrow conversion.

Upon close examination, a target enhancement pattern of larger lesions on contrast-enhanced T1WI was consistently observed in the IESL. Peripheral rim enhancement has been reported by Tang et al.^32^ in osteonecrosis, whereas the target enhancing pattern has not been described. We suggest that the reparative process of the outer vascular bone marrow to the inner ischemic insult contributes to this layered enhancement. We also hypothesize that peripheral enhancement is related to red marrow regeneration and that the central enhancing foci are composed of a fatty focus^25,34^.

Our study has limitations. First, it is a retrospective study with a modest number of cases. Second, all IESLs were found in pediatric patients with MPBT; we could not find MPBT with SM on MRI in our institute for comparison. As mentioned previously, SM from MPBT is extremely rare, and their image characteristics are similar to those of extracranial malignant tumors^15,16,19–23^. Their similar imaging features support our nine SM patients being representative of all “true metastasis” when compared to IESL from MPBT. Further studies comparing IESL and SM from MPBT may be considered. Third, although not statistically significant, the IESL group had longer time intervals between the diagnosis of the primary tumor and the presence of spinal lesions. This finding is possibly related to the small number of cases given that one IESL patient had spinal lesions developed 17 years after the initial diagnosis of brain tumor, which is much longer than that noted in the other two IESL patients. Fourth, all IESL patients had received whole-spine irradiation, whereas none of the SM patients underwent prior spinal radiotherapy. Of note, whole-spine irradiation is commonly performed in MPBT due to the high incidence of neuroaxial metastasis; in contrast, spinal irradiation is not routinely performed in extracranial malignancy until the occurrence of spinal metastasis. Fifth, we did not analyze the difference between IESL and SM with advanced MRI techniques, such as dynamic contrast-enhanced T1WI, chemical-shift (in–out phase), diffusion-weighted imaging, and perfusion study^35,36^. Finally, we did not perform biopsies for all lesions with abnormal MRI signals, which may reflect the hesitancy of practitioners to avoid repeated invasive procedures in pediatric patients.

In conclusion, indolent enhancing spinal lesions of malignant primary brain tumors in pediatric patients are related to treatment-induced delayed bone marrow changes. We suggest that ischemic insult, such as bone infarction or necrosis, is the main mechanism involved. IESL in pediatric patients with malignant primary brain tumors can be differentiated from spinal metastasis by their imaging characteristics. We recommend close follow-up rather than aggressive investigation and treatment for these IESLs.

## Data Availability

The datasets generated and/or analyzed during the current study are available from the corresponding author on reasonable request.
